# Efficacy of 18F-2-fluoro-2-deoxy-D-glucose Positron Emission Tomography/Computerized Tomography for Bone Marrow Infiltration Assessment in the Initial Staging of Lymphoma

**DOI:** 10.4274/mirt.54376

**Published:** 2017-06-01

**Authors:** Ali Ozan Öner, Evrim Sürer Budak, Funda Aydın, Ozan Salim, Orhan Kemal Yücel, Bahar Akkaya, Tayfur Toptaş, Adil Boz, Akın Yıldız, Fırat Güngör, Levent Undar

**Affiliations:** 1 Afyon Kocatepe University Faculty of Medicine, Department of Nuclear Medicine, Afyonkarahisar, Turkey; 2 Antalya Training and Research Hospital, Clinic of Nuclear Medicine, Antalya, Turkey; 3 Akdeniz University Faculty of Medicine, Department of Nuclear Medicine, Antalya, Turkey; 4 Akdeniz University Faculty of Medicine, Department of Hematology, Antalya, Turkey; 5 Akdeniz University Faculty of Medicine, Department of Pathology, Antalya, Turkey; 6 Marmara University Faculty of Medicine, Department of Hematology, İstanbul, Turkey

**Keywords:** Hodgkin’s lymphoma, non-Hodgkin’s lymphoma, positron emission tomography/computerized tomography, Bone marrow biopsy

## Abstract

**Objective:**

Currently ^18^F-2-fluoro-2-deoxy-D-glucose (^18^F-FDG) positron emission tomography/computerized tomography (PET/CT) is being successfully used for staging and follow-up of Hodgkin’s lymphoma (HL) and non-Hodgkin’s lymphoma (NHL). Various studies have demonstrated that PET/CT effectively detects bone marrow involvement (BMI) and is concordant with bone marrow biopsy (BMB) findings, thus it is deemed as a complementary method. This study was aimed to evaluate ^18^F-FDG-PET/CT efficiency for detection of BMI in HL and NHL.

**Methods:**

The study included 172 lymphoma cases who were admitted to Akdeniz University Medical School Department of Nuclear Medicine for initial staging with PET/CT. Visual and semiquantitative assessments were performed for PET/CT scan findings of the cases. The maximum standard uptake (SUV_max_) value was the quantitative parameter used for ^18^F-FDG-PET scan. In visual assessment, bone marrow metabolic activity that is greater than the liver was considered as pathologic. For semiquantitative assessment, regions of interest were drawn for SUV_max_ estimation, which included iliac crest in cases with diffusely increased metabolic activity and the highest activity area in cases with focal involvement. BMB was considered as the reference test.

**Results:**

On visual assessment of all the cases, PET/CT was found to yield 31% sensitivity and 85% specificity rate for detection of BMI. On visual assessment of HL cases, sensitivity rate was determined as 80%, and specificity as 78%, while in NHL cases the corresponding values were 24% and 90%, respectively. On semiquantitative assessment of HL cases, considering SUV_max_≥4, sensitivity was found as 80% and specificity as 68%. In NHL patients, considering SUV_max_≥3.2, sensitivity rate was detected as 65% and specificity as 58%.

**Conclusion:**

In this study, a moderately high concordance was observed between PET/CT and BMB findings. PET/CT appears to be a significant method for detecting BMI. Although PET/CT is not a substitute for BMB, we suggest it can be used as a guide to biopsy site and a complementary imaging technique for BMB.

## Abstract (tr)

**Amaç:**

Günümüzde ^18^F-2-fluoro-2-deoksi-D-glukoz (^18^F-FDG) pozitron emisyon tomografisi/bilgisayarlı tomografi (PET/BT), Hodgkin lenfoma (HL) ve non-Hodgkin lenfomaların (NHL) evrelemesinde ve takibinde başarılı bir şekilde kullanılmaktadır. Değişik çalışmalarda PET/BT’nin kemik iliği tutulumunu göstermede etkinliği ve kemik iliği biyopsisi (KİB) ile uyumu gösterilmiş ve tamamlayıcı yöntem olarak kullanılması önerilmiştir. Biz de bu çalışmada HL ve NHL’de kemik iliği infiltrasyonunu değerlendirmede PET/BT’nin etkinliğini değerlendirmeyi amaçladık.

**Yöntem:**

Çalışmaya Akdeniz Üniversitesi Tıp Fakültesi Nükleer Tıp Anabilim Dalı’nda başlangıç evreleme için PET/BT tetkiki yapılan 172 lenfoma olgusu dahil edilmiştir. Olguların PET/BT tetkikinde görsel ve semikantitatif değerlendirme yapıldı.^18^F-FDG-PET tetkikinde kantitatif parametre olarak maksimum standart uptake değeri (SUV_maks_) kullanıldı. Görsel değerlendirmede karaciğerden daha yüksek metabolik aktivite gösteren kemik iliği aktivitesi patolojik olarak değerlendirildi. Semikantitatif değerlendirmede ise kemik iliğinde diffüz metabolik aktivite artışı gösteren olgularda iliak kanattan, fokal tutulumda ise en yüksek aktivite gösteren alandan ilgi alanı çizdirilerek SUV_maks_ değeri hesaplandı. KİB sonuçları referans olarak alınmıştır.

**Bulgular:**

Tüm olgular için görsel değerlendirmede PET/BT’nin kemik iliği infiltrasyonunu göstermede duyarlılığı %31, özgüllüğü %85 olarak saptanmıştır. HL olgularında görsel değerlendirmede duyarlılık %80, özgüllük %78; NHL olgularında ise duyarlılık %24, özgüllük %90 olarak bulunmuştur. Semikantitatif değerlendirmede HL’de SUV_maks_≥4 alındığında duyarlılık %80, özgüllük %68 olarak saptanmıştır. NHL’de ise SUV_maks_≥3,2 olarak belirlendiğinde duyarlılık %65, özgüllük %58 olarak bulunmuştur.

**Sonuç:**

Bu çalışmada, çok yüksek olmamakla birlikte, PET/BT bulguları ile KİB sonuçları arasında uyum izlendi. PET/BT görüntülemenin kemik iliği infiltrasyonu olup olmadığını göstermede etkili bir yöntem olduğunu görmekteyiz. PET/BT’nin, KİB’nin yerini almamakla birlikte, biyopsi yapılabilecek bölgeyi göstermede yönlendirici ve KİB’yi tamamlayıcı bir görüntüleme yöntemi olarak kullanılabileceğini düşünmekteyiz.

## INTRODUCTION

Accurate staging of lymphomas is essential both to implement effective treatment protocols and minimize side effects (1). Identification of bone marrow infiltration (BMI) has an important role in staging (2). Bone marrow involvement indicates generalized disease in lymphoma patients, and the standard method established for its evaluation is bone marrow biopsy (BMB). BMB from unilateral iliac crest is the routine first-line method used for staging (3,4). However, this method has certain limitations since it is an invasive method and allows for evaluation of a limited part of the bone marrow. BMI can also be detected by imaging techniques. Computerized tomography (CT) detects cortical bone lesions and late stage bone changes. However, it has a low sensitivity rate for detecting early stage changes (5). Magnetic resonance (MR) is not used in routine practice since it is a sensitive but costly technique, which needs longer imaging time and is anatomically limited.

Currently, ^18^F-2-fluoro-2-deoxy-D-glucose (^18^F-FDG) positron emission tomography (PET/CT) is being successfully used for both staging and follow-up of Hodgkin’s lymphoma (HL) and non-Hodgkin’s lymphoma (NHL) (2,6,7). Various studies have demonstrated that PET/CT effectively detects bone marrow involvement and is concordant with BMB findings (1,5,8). Thus, it is deemed as a complementary method (9). This study aimed to evaluate the efficacy of ^18^F-FDG-PET/CT in detection of BM infiltration in HL and NHL.

## MATERIALS AND METHODS

### Patients

This study, approved by the Akdeniz University Medical School Local Ethics Committee, included histopathologically confirmed, treatment naïve 172 lymphoma cases (50 F, 122 M; age interval 3-85; mean age 45.37±21.14; 64 HL, 108 NHL) who underwent initial staging with PET/CT at Akdeniz University Medical School Department of Nuclear Medicine between July 2009 and December 2013. Patients included in the study did not have other concomitant malignancies. Additionally, patients did not receive any bone marrow stimulation therapy before PET/CT scanning. The maximum interval between PET/CT scan and BMB was 10 days.

### Positron Emission Tomography/Computerized Tomography Scanning

Intravenous 0.1 mCi/kg ^18^F-FDG was administered to each patient following 6 hours of fasting, with a blood glucose level below 200 mg/dL. The intravenous/oral contrast agent was administered. After 45-60 minutes of waiting period, PET/CT images were acquired from the vertex to the upper thigh with Siemens Biograph True Point PET/CT scanner (CT section thickness 3 mm, 110 mAs, 120 kV; 3 minutes per-bed PET) (Siemens, Erlangen, Germany) at the PET/CT unit. Attenuation corrected PET, CT and fusion PET/CT images were reviewed simultaneously; visual and semiquantitative assessments were performed. The maximum standard uptake value (SUV_max_) was the quantitative parameter used for ^18^F-FDG-PET scan. In visual assessment, bone marrow metabolic activity that is greater than the liver was considered to be pathologic. For semiquantitative assessment, regions of interest (ROI) were drawn for SUV_max_ estimation, which included iliac crest in cases with diffusely increased metabolic activity and the highest activity area in cases with focal involvement.

### Bone Marrow Biopsy

Unilateral BMB of the posterior iliac crest was performed by different hematologists as part of routine clinical evaluation, and the presence of marrow infiltration was interpreted by an experienced hematopathologist who was blinded to the PET/CT results. Trephine biopsy samples were analyzed following the standard procedures. BMB was considered as positive in the presence of lymphoma involvement. Although flow cytometric immunophenotyping of marrow aspirates can be performed, this method was not used for the diagnosis of bone marrow involvement.

### Data Analysis

BM biopsy results were regarded as the reference test for evaluating BMI.

Cases with concordant findings in both PET/CT and BMB (both positive or negative) were evaluated as true positive or true negative results. Non-concordance between these two parameters was described as false negativity or false positivity.

The sensitivity and specificity rates, positive predictive value (PPV) and negative predictive value (NPV) of PET/CT for detecting BM infiltration were determined for all cases. Additionally, receiver operating characteristics (ROC) curves were formed to determine cut-off values for SUV_max_. Analyzes were performed with PASW 18 (SPSS/IBM, Chicago, IL, USA) software.

## RESULTS

Among the 172 cases, BMI was detected by PET/CT in 33 (19.1%) and by BMB in 42 (24.4%) patients (Table 1). Among the 33 cases with infiltration on PET/CT, 11 had diffuse heterogeneous patchy accumulations while 22 had unifocal/multifocal accumulations. Within the 64 HL patients, BMI was detected by PET/CT in 17 (26.5%) and by BMB in 5 (7.8%) (Table 2), while among the 108 NHL patients, BMI was detected by PET/CT in 16 (14.8%) and by BMB in 37 (34.2%) (Table 3).

Concordance between PET/CT and BMB was observed in 123 (71%) of 172 patients. Both tests were negative in 110 patients and both were reported positive in 13 patients. A concordance between PET/CT and BMB was detected in 50 (78%) HL patients, both tests were negative in 46 and positive in 4 patients. Non-concordance was observed in 14 (22%) of these patients, 13 patients were positive on PET/CT but negative on BMB while 1 patient was positive on BMB but negative on PET/CT. Concordance was detected in 73 (67%) NHL patients, both tests were negative in 64 and positive in 9 patients. The tests were non-concordant in 35 patients, 7 patients were positive on PET/CT but negative on BMB while 28 patients were positive on BMB but negative on PET/CT.

On visual assessment of all the cases, PET/CT was found to have a 31% sensitivity and 85% specificity rate for detection of BMI with 39% PPV and 79% NPV (Table 4). Visual assessment of HL cases showed 80% sensitivity, 78% specificity with 24% PPV and 98% NPV (Table 4), while in NHL cases the corresponding values were 24%, 90%, 56% and 70%, respectively (Table 4).

Semiquantitative assessment was performed using SUVmax values and ROC curves based on BMB findings. Area under the ROC curve (AUC) estimated for all patients was 0.6386, that of HL patients was 0.7763 and NHL patients was 0.6534. Cut-off, sensitivity and specificity values were estimated using ROC curves for all patients and HL and NHL patient subgroups. The results were as follows, respectively; cut-off 3.5, 4, 3.2; sensitivity 59%, 80%, 65%; specificity 62%, 68%, 58% (Table 5).

Estimated mean SUVmax value was 12.02 g/mL for the 33 patients with positive findings on PET/CT; 11.67 g/mL for 13 patients who were positive on both PET/CT and BMB; and 12.25 g/mL for 20 patients who were positive on PET/CT but negative on BMB (Table 6).

## DISCUSSION

Initial evaluation including determination of anatomic distribution of the disease extent is an essential factor to predict both disease-free and overall survival in lymphoma patients. BM involvement in lymphoma indicates generalized disease and is a predictor of poor prognosis. Besides the role of BMI in primary staging, it has a specific clinical significance for guiding the treatment approach (2). In routine clinical practice, BMB is used to evaluate BM involvement. Although BMB is primarily a safe and risk-free procedure, complications such as bleeding or infection can rarely occur. Additionally, being an invasive and painful procedure can be a disadvantage for patients. In case of insufficient sampling, repeated biopsies may be required. There is no consensus on whether BMB should be performed uni- or bi-laterally, thus biopsies are usually obtained from unilateral iliac crest blindly, which may lead to high false negative rates. In light of the mentioned reasons, there is growing interest in the search for accurate non-invasive methods to evaluate BMI. ^18^F-FDG-PET/CT is a hybrid imaging technique used for primary staging, evaluating treatment response, re-staging and for follow-up after complete remission in lymphoma patients. Although, due to the limited number of studies, its role in evaluating bone/BM involvement is not well established, available results in the literature are promising (5,8).

Studies in the literature include mixed populations involving both HL and NHL patients. Cortés-Romera et al. (10) evaluated ^18^F-FDG-PET/CT performance for detecting BMI with reference to BMB in their study on 147 patients, comprising of 84 diffuse large B-cell lymphoma (DLBCL) and 63 HL patients. This study showed concordance between the two tests in 128 patients (87%) (74 DLBCL, 54 HL). Among these, both tests were reported positive in 21 and negative in 107 patients. Non-concordance was observed in 19 (14%) patients, 18 of which had negative BMB results although involvement was detected on ^18^F-FDG-PET/CT, indicating BMB was not obtained from active involvement sites. As a result of this study, ^18^F-FDG-PET/CT was reported to be 95% sensitive, 86% specific with 87% accuracy, 54% PPV and 99% NPV for detecting BMI. It was concluded that ^18^F-FDG-PET/CT had higher BMI detection rates in DLBCL and HL patients and in serving as a guide to biopsy sites, and that it can be used as a supplement to BMB (10).

In our study, among the 172 patients, 64 were diagnosed as HL and 108 as NHL. Thirty-three (19.1%) patients were found positive by ^18^F-FDG-PET/CT and 42 (24.4%) were positive on BMB. Concordance between the two tests was observed in 123 of the 172 patients (71%). Both tests were reported negative in 110 patients and both were positive in 13. Based on the total study population in the current study, ^18^F-FDG-PET/CT was detected to have 31% sensitivity and 85% specificity rate in detecting BMI with 39% PPV and 79% NPV. Additionally, AUC and cut-off values for the whole study population were 0.6386 and 3.5, respectively.

Similar studies on separate groups of HL and NHL patients are also available in the literature. In the study by Muzahir et al. (11), BMB from bilateral iliac crests were obtained from 122 HL patients and ^18^F-FDG-PET/CT findings were compared to these results that are considered as the gold standard. Accordingly, ^18^F-FDG-PET/CT was found to be 100% sensitive, 76.57% specific for detecting BMI in HL patients, with 78.62% diagnostic accuracy, 76.57% NPV and 29.72% PPV. The high sensitivity of ^18^F-FDG-PET/CT in this study was attributed to the positive ^18^F-FDG-PET/CT results in all of the BMB positive patients (11).

In the meta-analysis by Cheng and Alavi (12) including 7 studies comprising a total of 687 HL patients, ^18^F-FDG-PET/CT was found superior to BMB in detecting BMI. Pooled sensitivity of ^18^F-FDG-PET/CT was determined as 94.5% [95% confidence interval (CI): 89.0-97.8%] whereas the corresponding estimate for BMB was 39.4% (95% CI: 30.8-48.8%).

In the study by Adams et al. (13) on 26 newly diagnosed HL patients, visual ^18^F-FDG-PET/CT results were compared to BMB of the right iliac crest, which is used as the gold standard method. Accordingly, ^18^F-FDG-PET/CT was found to be 100% sensitive (95% CI: 51.1-100%), 100% specific (95% CI: 81.8-100%) in detecting BMI with 100% (95% CI: 51.1-100%) PPV, 100% (95% CI: 81.8-100%) NPV. Additionally, SUV_max_ of BMB positive patients [mean±standard deviation (SD): 3.4±0.85] was higher than that of BMB negative patients almost reaching statistical significance (mean±SD 2.7±0.63) (p=0.052) (13).

In our study, 64 of the 172 patients were diagnosed with HL. Concordance between BMB and ^18^F-FDG-PET/CT was observed in 50 (78%) of the 64 patients. Among these 50 cases, both tests were negative in 46 and positive in 4. In the remaining 14 patients, 13 were ^18^F-FDG-PET/CT positive, BMB negative and one patient was BMB positive without any involvement on ^18^F-FDG-PET/CT. Thus, considering BMB as the gold standard, ^18^F-FDG-PET/CT was found to be 80% sensitive, 78% specific for detecting BMI with 24% PPV and 98% NPV. The high sensitivity and specificity values were consistent with the literature. On semi-quantitative assessment, there was no significant difference in SUV_max_ values between BMB positive and negative cases among the PET/CT positive patients. Additionally, in our study, AUC and cut-off values for HL patients were found as 0.7763 and 4, respectively.

Similar studies on NHL patients are also available in the literature. In the study by Muslimani et al. (14), 97 NHL patients were grouped according to the presence of low or high grade disease, and the results of ^18^F-FDG-PET/CT scan for initial staging and unilateral iliac crest BMB were compared. Unlike other studies in the literature, samples were obtained from the involvement sites in BMB-negative patients with ^18^F-FDG-PET/CT images suggesting BMI. Consequently, BMB from sites of involvement of the 11 patients who were initially BMB-negative and ^18^F-FDG-PET/CT positive, revealed 6 positive BMB results. Positive repeat biopsies were obtained from the contralateral iliac crest in 1, from the humerus in 2, from the tibia in 1 and from the fourth vertebra in one patient. Thus, ^18^F-FDG-PET/CT was 79% sensitive, 91% specific for detecting BMI with 87% PPV and 87% NPV. Additionally, there was no significant difference between the low and high grade NHL groups in terms of the ability of ^18^F-FDG-PET to detect BMI (sensitivity p=0.23, specificity p=0.64). In conclusion, the high potential of ^18^F-FDG-PET in detecting BMI in NHL was highlighted and BMB sampling was recommended for BMB negative patients whose ^18^F-FDG-PET scan demonstrates BM involvement (14). In our study, biopsies were obtained only from the iliac crest and not from other sites observed positive on PET/CT, which is a limitation of our study. We could have found higher sensitivity and specificity values if biopsy sampling was done from sites other than the iliac crest in cases who were biopsy negative. Many studies in the literature have reported that in the majority of BMB-negative cases multifocal involvement was observed on ^18^F-FDG-PET/CT and that biopsies obtained from the sites of involvement were almost always positive (5,15,16,17).

In our study, among the 33 cases with infiltration on PET/CT, 11 had diffuse heterogeneous patchy accumulations. Diffuse accumulations may be secondary to benign conditions such as inflammation, thus some studies (13) have excluded such cases while others have not (11). In our study diffuse accumulations were heterogeneous and patchy, thus they were included since they were not homogenous lesions.

In the study by Lee et al. (18), 120 high grade NHL patients comprised of newly diagnosed DLBCL and peripheral T-cell lymphoma cases were included to assess the role of ^18^F-FDG-PET/CT in the detection of BMI. Bilateral iliac crest BMB results were considered the gold standard. ^18^F-FDG-PET/CT and BMB results were concordant in 100 of the 120 patients (both positive or negative) while 20 were non-concordant. Besides, SUV_max_ values of patients demonstrating abnormal ^18^F-FDG accumulation were significantly higher as compared to those with normal ^18^F-FDG accumulation. It was concluded that ^18^F-FDG-PET/CT and BMB are complementary techniques in assessing BMI in patients with high-grade lymphomas, and obtaining biopsies from sites of accumulation was recommended for patients showing ^18^F-FDG-avidity although standard iliac crest BMB are negative (18).

Berthet et al. (19) evaluated ^18^F-FDG-PET/CT performance for detecting BMI with reference to BMB and its effect on progression-free/overall survival in their study on 142 patients with DLBCL. In case of negative BMB, ^18^F-FDG-PET/CT accumulation areas were evaluated by biopsy or MR images. Accordingly, as compared to BMB, ^18^F-FDG-PET/CT had significantly higher sensitivity (94% vs. 24%, p<0.001), NPV (98% vs. 80%) and accuracy (98% vs. 81%). Multivariate analysis showed that BMI detected by ^18^F-FDG-PET/CT was an independent predictor of progression-free survival (PFS) (p=0.02) but not for overall survival. It was concluded that, assessment of BMI with ^18^F-FDG-PET/CT has higher diagnostic and prognostic prediction in newly diagnosed DLBCL patients as compared to BMB (19).

Zhou et al. (20) evaluated the role of ^18^F-FDG-PET/CT in detecting BMI and compared overall (OS) and PFS rates of patients who were concordantly negative (PET-CT/BMB-) or positive (PET-CT/BMB+) in 55 patients with newly diagnosed extranodal natural killer/T cell lymphoma. Using BMB results as reference, the study found the sensitivity and specificity rates of ^18^F-FDG-PET/CT for detecting BMI as 100% and 86%, respectively. Following the median follow-up period of 16 months (range, 3-43 months) PET-CT/BMB positive patient group showed worsened 2-year OS as compared to PET-CT/BMB negative group (84.8% vs. 67.9%, p<0.05). On the other hand, the estimated 2-year PFS rates for PET-CT/BMB negative and PET-CT/BMB positive patients were 72.7% and 41.9%, respectively. However, it was concluded that, due to the small number of PET-CT/BMB positive patients, it would be incorrect to conclude that survival rates were similar for both groups in advanced stage patients. Finally, the prognostic and complementary diagnostic role of ^18^F-FDG-PET/CT in detecting BMI, especially in cases missed by BMB, was underlined (20).

In our study, 108 of the 172 patients had NHL diagnosis and among these 16 (14.8%) were ^18^F-FDG-PET/CT positive and 37 (34.2%) were BMB positive. Subtyping/grading was not performed for NHL patient group. Concordance between the two tests was observed in 73 (67%) patients; 64 patients were PET-CT/BMB negative while 9 patients were PET-CT/BMB positive. Non-concordance was observed in 35 patients; of which 7 were ^18^F-FDG-PET/CT positive, BMB negative and the remaining 28 patients were BMB positive, ^18^F-FDG-PET/CT negative. As a result, ^18^F-FDG-PET/CT was found to be 24% sensitive, 90% specific for detecting BMI with PPV 56% and NPV 70%. We suggest that the low sensitivity for the NHL subgroup may be due to the lack of histological subgrouping in this patient group. Studies have reported higher ^18^F-FDG-PET/CT sensitivity rates in detecting BMI in aggressive NHL subtypes, while 2/3 false negativity ratio was observed in indolent histological forms (e.g., grade 1 and 2 follicular lymphomas) leading to lower sensitivity values (9). Additionally, in our study, AUC and cut-off values for NHL patients were found as 0.6534 and 3.2, respectively.

## CONCLUSION

In this study, a moderately high concordance (71%) was observed between PET/CT and BMB findings. The rate of concordance was higher in HL patients (78%) as compared to NHL patients (67%). In conclusion, PET/CT appears to be a significant method for detecting BM infiltration in comparison to BM biopsy which is an invasive method. Currently, BM biopsy is usually performed from the iliac crest while PET/CT has the advantage of whole body imaging to allow for detection of involvement sites other than the iliac bone. In this regard, although PET/CT is not a substitute for BM biopsy, we suggest that it can be used as a guide to biopsy site and a complementary imaging technique for BM biopsy.

## Figures and Tables

**Table 1:**
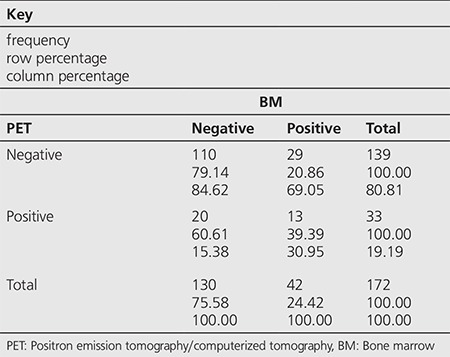
Distribution of the patients according to positron emission tomography/computerized tomography and bone marrow biopsy test results

**Table 2:**
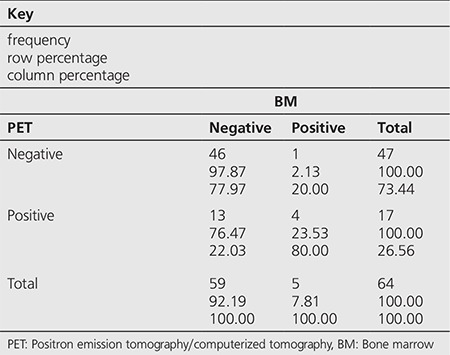
Distribution of the Hodgkin’s lymphoma patients according to positron emission tomography/computerized tomography and bone marrow biopsy test results

**Table 3:**
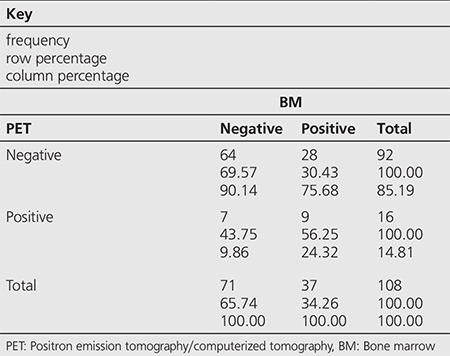
Distribution of the non-Hodgkin’s lymphoma patients according to positron emission tomography/computerized tomography and bone marrow biopsy test results

**Table 4:**
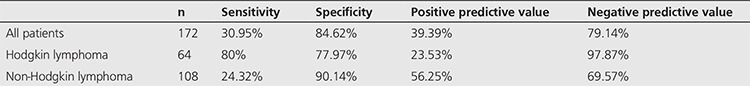
Sensitivity, specificity, positive predictive value and negative predictive value (95% confidence interval) of positron emission tomography/computerized tomography with respect to bone marrow biopsy in the whole population and Hodgkin’s lymphoma and non-Hodgkin’s lymphoma subgroups

**Table 5:**
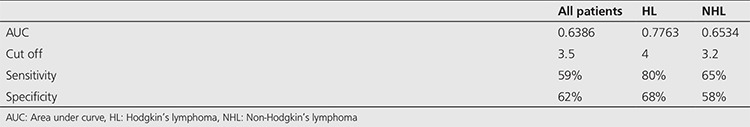
Receiver operating characteristics estimated area under curve with 95% confidence intervals, cut off, sensitivity and specificity values for the whole population and for Hodgkin’s lymphoma and non-Hodgkin’s lymphoma subgroups

**Table 6:**
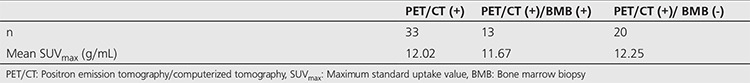
Numeric distribution and mean maximum standard uptake values for patients with positive results on positron emission tomography/computerized tomography
